# Multimodal magnetic resonance imaging on brain network in amnestic mild cognitive impairment: A mini-review

**DOI:** 10.1097/MD.0000000000034994

**Published:** 2023-08-25

**Authors:** Sheng-Lan Gao, Jinhuan Yue, Xiao-Ling Li, Ang Li, Dan-Na Cao, Sheng-Wang Han, Ze-Yi Wei, Guanhu Yang, Qinhong Zhang

**Affiliations:** a Graduate School of Heilongjiang University of Chinese Medicine, Harbin, China; b Shenzhen Frontiers in Chinese Medicine Research Co., Ltd., Shenzhen, China; c Division of CT and MRI, First Affiliated Hospital of Heilongjiang University of Chinese Medicine, Harbin, China; d Sanofi-Aventis China Investment Co., Ltd, Beijing, China; e Third Ward of Rehabilitation Department, Second Affiliated Hospital of Heilongjiang University of Chinese Medicine, Harbin, China; f Department of Specialty Medicine, Ohio University, Athens, OH.

**Keywords:** amnestic mild cognitive impairment, functional network, magnetic resonance imaging, multimodal, structural network

## Abstract

Amnestic mild cognitive impairment (aMCI) is a stage between normal aging and Alzheimer disease (AD) where individuals experience a noticeable decline in memory that is greater than what is expected with normal aging, but dose not meet the clinical criteria for AD. This stage is considered a transitional phase that puts individuals at a high risk for developing AD. It is crucial to intervene during this stage to reduce the changes of AD development. Recently, advanced multimodal magnetic resonance imaging techniques have been used to study the brain structure and functional networks in individuals with aMCI. Through the use of structural magnetic resonance imaging, diffusion tensor imaging, and functional magnetic resonance imaging, abnormalities in certain brain regions have been observed in individuals with aMCI. Specifically, the default mode network, salience network, and executive control network have been found to show abnormalities in both structure and function. This review aims to provide a comprehensive understanding of the brain structure and functional networks associated with aMCI. By analyzing the existing literature on multimodal magnetic resonance imaging and aMCI, this study seeks to uncover potential biomarkers and gain insight into the underlying pathogenesis of aMCI. This knowledge can then guide the development of future treatments and interventions to delay or prevent the progression of aMCI to AD.

## 1. Introduction

Alzheimer disease (AD) is a common, progressive, and fatal neurodegenerative disease that affects the brain. It is currently on the rise worldwide, becoming increasingly prevalent and accounting for approximately 80% of all cases of dementia.^[1,2]^ AD is characterized by a gradual decline in independent thinking and daily activities. It is a complex and irreversible neurologic disease that causes cognitive impairment.^[3,4]^ Amnestic mild cognitive impairment (aMCI) is a condition that is typically marked by memory problems that go beyond what is expected with normal aging. However, it dose not meet the clinical criteria for a diagnosis of AD. Instead, aMCI is often considered a transitional state, or a precursor, to the development of AD.^[5,6]^ There are 2 main subtypes of aMCI, namely single-domain aMCI (aMCI-s) and multidomain aMCI (aMCI-m).^[7]^ The former involves damage primarily to the memory domain, while the latter includes damage to the memory domain as well as at least one other cognitive domain. Early detection and intervention for aMCI are crucial in order to delay its progression to AD.^[8]^ Therefore, there is significant interest in the use of multimodal MRI techniques to study aMCI.

These techniques involve the use of different magnetic resonance imaging (MRI) scans, such as structural magnetic resonance imaging (sMRI), diffusion tensor imaging (DTI), and functional magnetic resonance imaging (fMRI), to gather information about the structure and function of the brain. By analyzing the existing literature on aMCI and using these multimodal MRI techniques, researchers hope to gain a deeper understanding of the underlying causes and mechanisms of aMCI. Furthermore, this research aims to identify potential biomarkers that can aid in the diagnosis and monitoring of aMCI.

## 2. Brain structural network study of aMCI

### 2.1. sMRI

sMRI has emerged as a valuable tool in investigating the local brain morphology in individuals with aMCI. This imaging technique not only helps us understand the overall brain structural networks, but also to provide a foundation for exploring the intrinsic connection between brain structure and function. Researchers primarily rely on voxel-based morphometry analysis to analyze sMRI data in aMCI studies. For instance, a study conducted by Zhang et al surveyed VBM studies on aMCI published up until June 2020.^[9]^ In their review, they found that individuals with aMCI exhibited significant gray matter atrophy in the left amygdala and right hippocampus. These brain regions are known to be involved in emotion, cognition, and perception. Furthermore, a meta-regression analysis showed that gray matter atrophy in the left inferior frontal gyrus and left angular gyrus was strongly associated with cognitive impairment individuals with aMCI. These findings provide valuable insight into potential biomarkers that can predict and diagnose aMCI. In another investigation, Sun et al delved into the differences in cortical thickness between individuals with AD, aMCI, and normal controls. They specifically focused on 2 subtypes of aMCI-m and aMCI-s.^[10]^ Their findings indicated that the average cortical thickness of the left insula, temporo-central gyrus, and right insula progressively decreased from healthy individuals to aMCI-s and then to aMCI-m. This suggests that aMCI-m is more likely to progress to AD, possibly serving as a transitional stage between aMCI-s and AD. Du et al explored the structural covariance networks of the anterior and posterior hippocampal regions in relation to verbal and spatial memory using partial least squares.^[11]^ They identified 3 distinct structural covariance network patterns: an age pattern, a specific aMCI pattern, and a spatial memory pattern. Importantly, individuals with aMCI displayed more extensive and severe damage in all 3 patterns, which was associated with a decline in verbal memory ability. This findings offers a potential neurobiological explanation for the conversion of aMCI and non-amnestic MCI into different types of dementia in the future. Moreover, previous studies have highlighted the utility of sMRI-based brain networks, tracking brain structural changes during aging, and investigating neurodegenerative diseases. These findings open avenues for providing new models and approaches to further comprehend these complex disorders.^[12,13]^

### 2.2. DTI

DTI is a type of imaging modality that does not require invasive procedures and is developed based on DWI. It is primarily used for studying injuries to the white matter fiber bundle in the brain.^[14]^ DTI involves analyzing parameters such as mean diffusivity and fractional anisotropy. In a study conducted by Fang et al, the researchers used graph theory analysis to construct structural brain networks of patients with AD, aMCI, and normal controls. The aim was to examine the changes in the brain network structure from 3 perspectives: global topology, edge characteristics, and node characteristics.^[15]^ Interestingly, all 3 groups displayed prominent, efficient, small-world characteristics in their brain networks. However, the fractional anisotropy-weighted brain networks of patients with AD and aMCI exhibited longer characteristic path lengths, which suggested global integration dysfunction. Moreover, abnormal characteristic path lengths were found to have a negatively correlation with clinical scores for cognitive impairment. This indicates that the extent of the disruption in brain network integration is associated with the severity of cognitive impairment in AD patients. In another study by Yan et al DTI data was collected from various groups including normal control participants, individuals with SCD, aMCI patients, and patients with d-AD.^[16]^ The aim was to analyze the Rich-club organizational structure. Surprisingly, all patient groups (SCD, aMCI, and d-AD) showed similar patterns of peripheral regional disconnection and reduced connectivity involving these regions. This suggests that peripheral region disruption may contribute to the decline in cognitive abilities, which can be considered as an early marker of subjective cognitive decline. These findings highlight the disruption patterns in the connectome of AD patients and provide evidence that the disruption of the rich club organization is a crucial factor in the progression of AD. Additionally, these disruptions can dynamically reflect the progression of the disease, making them valuable markers for monitoring and studying AD.

## 3. Brain functional network studies of aMCI

FMRI is a powerful and noninvasive technique that allows us to map the activity of different brain regions in the brain and examine how these regions interact with each other to form functional brain networks.^[17]^ Unlike task-based fRMI techniques, resting-state fMRI (rs-fMRI) does not require subjects to perform specific tasks and instead measures the spontaneous activity of the brain.^[18]^ Rs-fMRI techniques, such as low-frequency amplitude (amplitude of low-frequency fluctuations, ALFF), regional homogeneity, and functional connectivity (FC), have been widely used to study brain networks.^[19]^ In recent years, there has been a growing interest in using rs-fMRI to investigate brain functional networks.^[20,21]^ Xue et al conducted a study using rs-fMRI combined with graph analysis and found that the topological structure of brain FC networks was disrupted in individuals with SCD and aMCI compared to healthy controls.^[22]^ By examining the connections between rich and diverse clubs in the brain, the study identified 3 types of connections that may be useful for diagnosing the aMCI group and distinguishing between the SCD and aMCI groups. The researchers also observed significant differences in node efficiency and shortest path length between overlapping nodes within the clubs, which could serve as important biomarkers for diagnosing the AD spectrum.

One specific brain network that has been extensively studied is the default mode network (DMN), which is associated with an “internal focus” state.^[23]^ The DMN is responsible for various cognitive processes such as prospective and autobiographical memory, rumination, social cognition, evaluation, internal mental awareness, and self-consciousness. Studying the DMN using rs-fMRI can provide valuable insights into the brain changes associated with aMCI. In summary, fMRI combined with resting-state techniques has shown great potential in investigating brain functional networks in individuals with aMCI. These studies have provided valuable information on the disruption of brain networks in aMCI and have identified potential biomarkers for the diagnosis and understanding of the AD spectrum. Additionally, studying the DMN using rs-fMRI offers insights into the cognitive processes affected in aMCI.^[23]^ In their study, Pagen et al explored the FC between the cerebellar DMN and the entire brain using seed-based analysis.^[24]^ They compared the results of healthy elderly individuals with those of patients diagnosed with aMCI. The findings revealed that patients with aMCI exhibited lower levels of anticorrelation between the cerebellar DMN and several regions within the DMN of the brain. More specifically, the reduced anticorrelation was observed between the cerebellar DMN and the medial prefrontal cortex. It was further identified that this decreased anticorrelation was associated with poorer memory performance in individuals with aMCI. These results imply a negative correlation between the cerebellar DMN and the DMN in the brain during rest among the elderly population. Moreover, the reduced anticorrelation suggests an impact on the regulatory role played by the cerebellum in cognitive function, particularly in the executive components of memory function in neurodegenerative diseases. Additionally, Menon et al introduced the concept of the “triple network model,” which encompasses the DMN, salience network (SN), and executive control network (ECN).^[25]^ These 3 core brain networks closely interact and play crucial roles in regulating human cognition and emotional states. The SN, primarily comprised of the dorsal anterior cingulate cortex and the fronto-insular cortex, is responsible for identifying the most salient stimuli in both internal and external environments to guide behavior.^[26,27]^

Building upon this concept, Song et al conducted a meta-analysis to explore specific functional alterations within the SN among individuals diagnosed with MCI and aMCI.^[28]^ In their study, the results showed a decrease in ALFF/fractional ALFF in specific brain regions, including the left superior temporal gyrus, insula, middle frontal gyrus, and precentral gyrus in aMCI. Furthermore, a decrease in regional homogeneity was observed in the anterior portion of the cingulate gyrus, indicating altered local neural synchronization in this region. Additionally, the study revealed abnormal functional interactions between the SN and other networks, such as the DMN and ECN, in the inferior frontal gyrus of aMCI patients. These findings provide novel insights into the prediction of progression from healthy cognition to MCI or aMCI and identify potential targets for interventions aimed at slowing down disease progression. Moreover, the researchers hypothesize that decreased FC within the DMN and increased FC within the ECN and SN might be characteristic features of aMCI patients during rs-fMRI.^[29]^ These findings have the potential to contribute to the development of imaging biomarkers for more accurate diagnoses of aMCI.

Based on the research using task-state fMRI,^[30]^ the study aimed to explore the specific changes in the topological structure of the brain’s DMN during memory tasks. Specifically, the focus was on individuals with aMCI. The study found that individuals with aMCI exhibited decreased FC within the DMN both before and after engaging in memory tasks. This implies that the communication and coordination between different brain regions within the DMN were compromised in aMCI individuals. Surprisingly, the study also revealed a significant increase in local efficiency within the DMN of aMCI patients. This suggests that despite the overall decline in FC, there was an enhanced ability of local brain regions to efficiently process information within the DMN. However, there was no significant improvement in global efficiency within the DMN of aMCI patients. This indicates that while individual brain regions within the DMN showed improved information processing, the overall integration and communication between different regions remained impaired. Importantly, these network measures were found to be correlated with cognitive performance. This implies that the alterations in FC and efficiency within the DMN are closely related to the cognitive deficits observed in aMCI individuals. Overall, the findings of this study provide evidence of early functional reorganization occurring within the DMN of individuals with aMCI. This reorganization involves the formation of a non-optimized regular configuration, indicating a deviation from the typical DMN structure seen in normal aging. By expanding our understanding of the dynamic functional reorganization of brain networks from normal aging to the AD continuum, these findings contribute to our knowledge of the neurobiological mechanisms underlying cognitive decline in aMCI.

## 4. Limitations

Currently, the research in this domain is facing several limitations. Firstly, there is a common reliance on single-modal imaging methods, which restricts the exploration of the intricate relationship between the structural and functional networks in the brain. To overcome this limitation, it is essential to utilize various modal neuroimaging data, such as fMRI, DTI, and positron emission tomography, to enhance the reliability and depth of the research findings. Secondly, there are constraints in the collection and analysis of imaging data. To address this issue, it is crucial to establish large-sample multicenter databases. By incorporating data from multiple research centers, the conclusions drawn from the analysis of these databases will have higher accuracy and validity. Furthermore, the development of standardized data collection and analysis protocols will ensure consistency and comparability across different studies, enhancing the overall quality of the research. Lastly, most of the experiments conducted so far are cross-sectional studies of the aMCI brain networks. This means that the investigations focus on a single time point and fail to capture the dynamic changes occurring in these networks over time. To gain a comprehensive understanding of the progression of aMCI, it is imperative to conduct longitudinal studies that track individuals over an extended period.

## 5. Summary

Multimodal MRI brain network analysis, which includes the examination of both brain structure and function, can provide valuable insights into the changes occurring in the brains of individuals with aMCI. Furthermore, studies have indicated that individuals with aMCI-m are more likely to progress to AD compared to those with aMCI-s. aMCI-m can be regarded as a transitional stage between the early stages of aMCI, where impairments are limited to a specific domain, and the development of AD. FC changes and interactions within the DMN, ECN, and SN have been identified as potential biological markers for aMCI. These findings were uncovered through the use of fMRI, a technique that allows researchers to detect changes in brain activity patterns. To establish reliable imaging evidence for the differential diagnosis and prognosis of aMCI, it is crucial to conduct longitudinal and in-depth observations of the disease over time. By closely monitoring the progression of aMCI through advanced imaging techniques, researchers can gather valuable information that can aid in the accurate diagnosis and prediction of outcomes for individuals with aMCI.

## Author contributions

**Conceptualization:** Sheng-Lan Gao, Jinhuan Yue, Xiao-Ling Li, Ang Li, Sheng-Wang Han, Ze-Yi Wei, Qinhong Zhang.

**Data curation**: Sheng-Lan Gao, Jinhuan Yue, Xiao-Ling Li, Qinhong Zhang.

**Resources:** Sheng-Lan Gao, Jinhuan Yue, Xiao-Ling Li, Sheng-Wang Han.

**Validation:** Sheng-Lan Gao, Jinhuan Yue, Xiao-Ling Li, Ang Li, Dan-Na Cao, Sheng-Wang Han, Ze-Yi Wei, Guanhu Yang, Qinhong Zhang.

**Visualization:** Sheng-Lan Gao, Jinhuan Yue, Xiao-Ling Li, Ang Li, Dan-Na Cao, Sheng-Wang Han, Ze-Yi Wei, Guanhu Yang, Qinhong Zhang.

**Writing – original draft:** Sheng-Lan Gao, Jinhuan Yue, Xiao-Ling Li, Ang Li, Dan-Na Cao, Sheng-Wang Han, Ze-Yi Wei, Guanhu Yang, Qinhong Zhang.

**Writing – review & editing:** Sheng-Lan Gao, Jinhuan Yue, Xiao-Ling Li, Ang Li, Dan-Na Cao, Sheng-Wang Han, Ze-Yi Wei, Guanhu Yang, Qinhong Zhang.

**Funding acquisition:** Xiao-Ling Li, Dan-Na Cao.

**Methodology:** Xiao-Ling Li, Ang Li.

**Investigation:** Guanhu Yang, Qinhong Zhang.

**Project administration:** Guanhu Yang, Qinhong Zhang.

**Supervision:** Guanhu Yang, Qinhong Zhang.
